# Korean clinical entity recognition from diagnosis text using BERT

**DOI:** 10.1186/s12911-020-01241-8

**Published:** 2020-09-30

**Authors:** Young-Min Kim, Tae-Hoon Lee

**Affiliations:** 1 Graduate School of Technology & Innovation Management, Hanyang University, 222 Wangsimni-ro, Seongdong-gu, Seoul, South Korea; 2 Division of Interdisciplinary Industrial Studies, Hanyang University, 222 Wangsimni-ro, Seongdong-gu, Seoul, South Korea

**Keywords:** Clinical entity recognition, BERT, Korean, Diagnosis text

## Abstract

**Background:**

While clinical entity recognition mostly aims at electronic health records (EHRs), there are also the demands of dealing with the other type of text data. Automatic medical diagnosis is an example of new applications using a different data source. In this work, we are interested in extracting Korean clinical entities from a new medical dataset, which is completely different from EHRs. The dataset is collected from an online QA site for medical diagnosis. Bidirectional Encoder Representations from Transformers (BERT), which is one of the best language representation models, is used to extract the entities.

**Results:**

A slightly modified version of BERT labeling strategy replaces the original labeling to enhance the separation of postpositions in Korean. A new clinical entity recognition dataset that we construct, as well as a standard NER dataset, have been used for the experiments. A pre-trained multilingual BERT model is used for the initialization of the entity recognition model. BERT significantly outperforms a character-level bidirectional LSTM-CRF, a benchmark model, in terms of all metrics. The micro-averaged precision, recall, and f1 of BERT are 0.83, 0.85 and 0.84, whereas that of bi-LSTM-CRF are 0.82, 0.79 and 0.81 respectively. The recall values of BERT are especially better than that of the other model. It can be interpreted that the trained BERT model could detect out of vocabulary (OOV) words better than bi-LSTM-CRF.

**Conclusions:**

The recently developed BERT and its WordPiece tokenization are effective for the Korean clinical entity recognition. The experiments using a new dataset constructed for the purpose and a standard NER dataset show the superiority of BERT compared to a state-of-the-art method. To the best of our knowledge, this work is one of the first studies dealing with clinical entity extraction from non-EHR data.

## Background

Clinical entity recognition traditionally aims at electronic health records (EHRs) [1] generated by healthcare providers. EHRs contain clinical information about patients including diagnoses, laboratory tests, clinical notes, etc [2]. The target entities are mostly technical terms precisely written by medical specialists. The medical problem, treatment, and test are typical entity types of these texts [3]. The extracted entities are fundamental to build clinical informatics applications [4]. Identification of patient cohorts, extraction of adverse drug events, and finding the relationships of drug-disease treatment are some of the applications [1, 5, 6].

While the above applications concern the traditional biomedical informatics, there is also the demand for extracting clinical entities for new applications from another domain. It is automatic medical diagnosis via a dialogue system. There is a recent work dealing with medical diagnosis data extracted from user-generated QA set [7]. Its goal is to build an automatic medical diagnosis system, which operates via a conversational process. The clinical entities expressed in the data are essential to construct the system. However, entity recognition is not the main concern of the work although it is necessary for the actual use. The authors focus on building a system by reinforcement learning. Considering the increasing interests in healthcare applications, this kind of expanded use of clinical entity extraction will be a valuable research theme.

In this paper, we are interested in extracting clinical named entities from a new medical dataset, which is completely different from EHRs. The novelty of the dataset mainly comes from the originality of the data source and the annotated entity types. The dataset is collected from an online QA site for medical diagnosis, and the target language is Korean. Three different entity types, which are essential for the diagnosis, are defined and annotated. We use a state-of-the-art NLP technique, Bidirectional Encoder Representations from Transformers (BERT) [8] for the entity extraction. We show BERT using WordPiece tokenization is effective for the domain-specific named entity recognition in Korean.

**Fig. 1:**
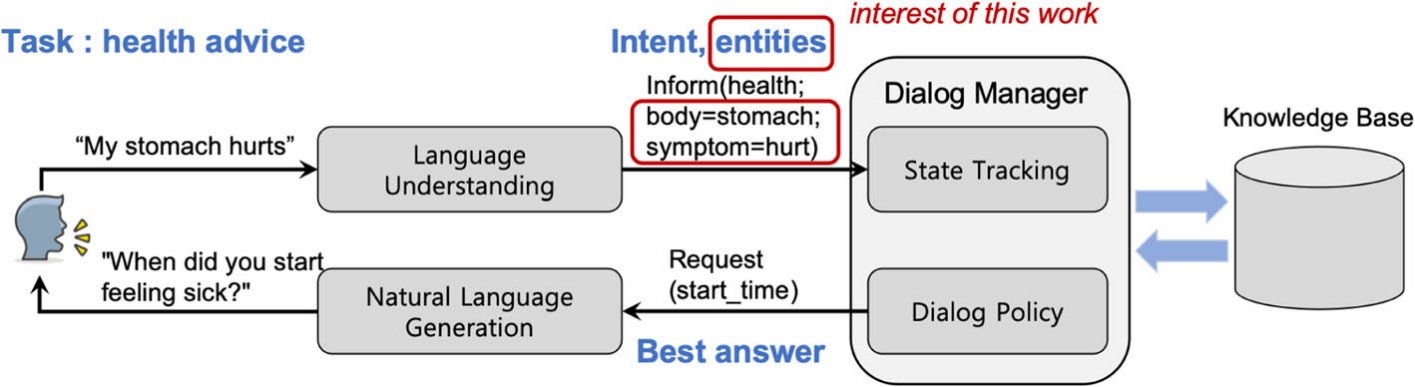
Architecture of a dialogue system

Our clinical entity recognition is originally designed to be a part of a dialogue system for health advice. The final goal of the system is to provide simple health advice to users via conversation. This work will be the first step for the final goal. Figure 1 shows the overall process of the target system following the recent dialogue system architectures [9–11]. The interest of this paper is emphasized with red rectangles.

From the initial user utterance, the system first extracts the user intent and useful entities. In the example, the extracted entities are stomach and hurt as body part and symptom respectively. Then a dialogue manager tries to understand the current state of the conversation to derive the best answer. In this case, the system tries to get the start time of the illness to exactly identify the current condition of the user. Here, the entity recognition is an important part that influences the most the final system performance.

Named entity recognition (NER) is usually considered as a sequence labeling task. The recent developments of long-short term memory (LSTM) variants such as bidirectional LSTM-CRF [12] and bi-LSTM-CNNs-CRF [13] have achieved a success in both NER and biomedical NER [14, 15]. Another popular research direction is to apply the attention mechanism [16]. Various sequence labeling approaches using the mechanism have been developed in different tasks, such as slot filling [17], role labeling [18], and bio-medical information detection [19].

The Korean NLP community has adopted similar methodologies for sequence labeling and also obtained better performances than traditional CRF-based approaches. However, because of the linguistic property that the words are not always clearly separated, the tokenization and input encoding influence a lot to the final performance in general. The character-level n-gram encoding with additional linguistic information is one of the state-of-the-art approaches for Korean NER [20]. A recent work reports that *jamo* (Korean alphabet) level representation extracts well the word semantics in terms of word similarity [21]. Another work on NER proposed to use a hybrid representation of morpheme vectors [22]. These works focus on finding the best input representation in common.

BERT is a recently developed language representation model. It trains a deep bidirectional representation of a large unlabeled corpus using stacked Transformer encoders. Then, the representation is fine-tuned with an additional output layer for downstream NLP tasks. The main difference with Embeddings from Language Models (ELMo) [23] and OpenAI Generative Pre-training Transformer (GPT) [24], the precedent models, is that BERT is bidirectional when applying self-attention.

In this work, we use BERT to train a NER model for medical entity recognition. We investigate the effects of proper tokenization as well as labeling strategy for evaluation. We empirically show the simple WordPiece representation is effective for the domain-specific NER in Korean even with a small dataset. An additional experiment is also provided to verify the effectiveness of BERT for Korean NER on a standard dataset. A bi-LSTM-CRF model is selected as a benchmark to show the superiority of BERT for Korean medical NER.

## Methods

We constructed a clinical NER dataset that contains medical experts’ diagnoses to the questions of an online QA service. BERT is applied to the dataset to extract the clinical entities. We slightly modified the BERT labels to separate the postpositions, which are very common in Korean. Different labeling formats for the evaluation are proposed to find the most effective one to assess the entity recognition results.

### Bidirectional encoder representations from transformers

**Fig. 2:**
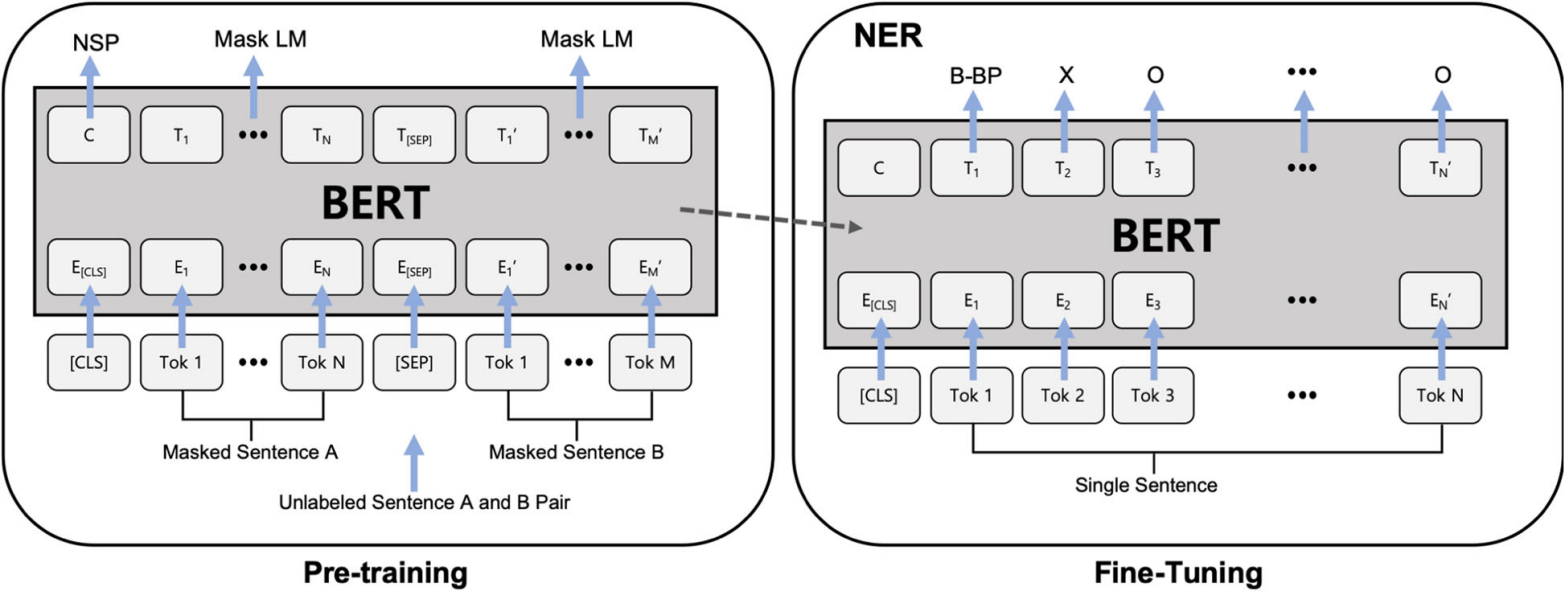
BERT architecture and fine-tuning

Figure 2 shows the architecture of BERT. The pre-trained model weights are re-used for the training of an NLP task, NER, in our case. Unlike the precedent models, BERT pre-trains the model using a masked language model (LM). While a standard LM aims to predict the next token for each token in a sentence, a masked LM predicts a randomly masked token given a sentence. This simple but effective mechanism allows the bi-directionality in the model. BERT produces surprisingly good results on most NLP tasks. It outperforms the existing state-of-the-art methods on 11 tasks including NER. An interesting part for us is that it uses a simple WordPiece tokenization [25] for input that is more appropriate for Korean than the other English text encodings.

**Fig. 3:**
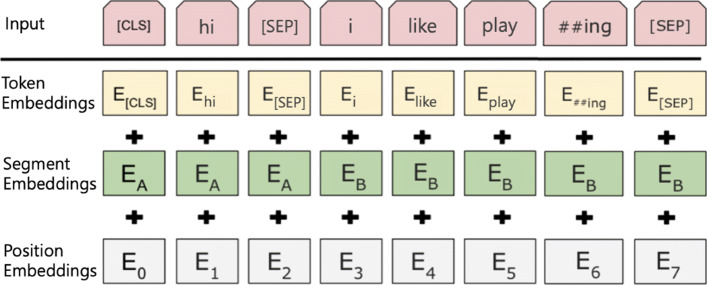
BERT input representation. (modified image from [8])

WordPiece tokenization separates words into different tokens as shown in the ‘input’ row of Fig. 3. Most meaningful words are kept and the other words are tokenized into pieces. To express the continuity of the tokens, two sharps (##) are attached in front of the token when it is a part of the precedent token. In the example, the word ‘playing’ is split into ‘play’ and ‘##ing’. It means the word was less occurring than the other words when training the WordPiece representation. The separated tokens are called sub-words. Two special tokens [CLS] and [SEP] are added to express the start of the data instance and the end of a sentence.

The segmentation embeddings are used to distinguish different sentences. The position embeddings represent the token positions in the instance. Each token is finally represented by the sum of the token embedding, the segmentation embedding, and the position embedding. With this tokenization, BERT keeps a very small number of sub-words. In English, the vocabulary size of a trained BERT model is only 30,522. The final input embedding dimension is 768.

BERT uses the encoders of the Transformer architecture. Using the Transformers has become very common in language modeling [26]. The Transformer has Multi-Head Attention, which concatenates *h* different attention layers with different initializations [16]. Multi-Head Attention is written as follows:

$$ \begin{aligned} Multihead(Q, K, V) = Concat(head_{1},..., head_{h})W^{O} \\ where~head_{i} = Attention\left(QW^{Q}_{i}, KW^{K}_{i}, VW^{V}_{i}\right) \end{aligned} $$

The *head*_*i*_ is *i*^*th*^ attention head. $\phantom {\dot {i}\!}W^{Q}_{i} \in \text {I\!R}^{d_{emb}\times d_{k}}, W^{K}_{i} \in \text {I\!R}^{d_{emb}\times d_{k}}, W^{V}_{i} \in \text {I\!R}^{d_{emb}\times d_{v}}$ and $\phantom {\dot {i}\!}W^{O} \in \text {I\!R}^{hd_{v}\times d_{emb}}$ are projection matrix parameters. *Q*, *K* and *V* are different input matrices. At the beginning, input matrix *X* are used for all three matrices. Then their projections $XW^{Q}_{i}, XW^{K}_{i}$ and $XW^{V}_{i}$ become *Q*_*i*_,*K*_*i*_, and *V*_*i*_. These matrices are used to compute the following Scaled Dot-Product Attention.

$$ \begin{aligned} Attention(Q, K, V) = softmax\left(\frac{QK^{T}}{\sqrt{d_{k}}}V\right) \end{aligned} $$

We use a pre-trained BERT model’s weights for the initialization of the clinical entity recognition task. The BERT architecture is reused and the input and output are adjusted to our task.

### Data collection and training set

The source data is collected from the biggest Korean online QA service^1^ that the platform is provided by a web portal. The questioners and answerers are all the portal users. The questions are categorized into different sections according to the nature of the question. In the medical section, there are 24 different departments. Each medical department has a special subcategory, which contains the QA pairs answered by medical specialists.

We select four departments most relevant for the automatic diagnosis. The department of neurology, neurosurgery, internal medicine, and otorhinolaryngology are those four. We collect QA data answered by medical specialists from Jan. 2009 to Aug. 2018. Two medical specialists who wrote most of the answers per department have been selected. Then QA sets answered by the selected specialists have been all collected.

Among the data, we randomly selected 200 QA pairs per department. To construct the training set, we first use the answers only because they include much more formulaic expressions than the questions. After testing the effectiveness of BERT for clinical entity recognition using answer sets, we also test question sets, which include much more colloquial expressions.

By filtering out some duplicated answers and unnecessary questions such as MRI reading or military service exemption issue, we have a total of 536 answers. As a long answer tends to include supplementary information at the end, we kept 5 first sentences the maximum excluding greeting messages. The statistics of the QA dataset is given in Table 1.

**Table 1:** Statistics of the QA dataset for diagnosis

**department**	**# anwsers**	**# sents**
neurology	126	630
neurosurgery	156	780
internal medicine	131	655
otorhinolaryngology	123	615
total	536	2189

For the construction of the training set, three annotators discussed the annotation guidelines. Each instance, which corresponds to an answer, is annotated by one annotator and is reviewed by another annotator. After a detailed review of the source data, we select three different clinical entity types that are essential for diagnosis. These are ‘disease’, ‘symptom’ and ‘body part’. The definition of the entity types is given in Table 2.

**Table 2:** Entity definition for clinical NER

**Entity type**	**definition**
Disease (DZ)	disease name. used for final diagnosis
Symptom (SX)	symptom which can be detected by users
Body Part (BP)	body part where the symptom reveals

For the annotation, we first collected the entity dictionaries from “National Health Information Portal” and hospital web sites. The number of terms is 2,191 for disease names, 142 for symptoms, and 139 for body parts. Some conflict terms have been preprocessed before annotation. Terms not found in the dictionaries have been searched using the other health information portals such as “Infectious Disease Portal” of Korea Centers for Disease Control and Prevention (KCDC) and “Medical Encyclopedia” of Seoul National University Hospital.

The main annotation guidelines for the training set construction are as follows:

- Frequent informal disease names such as “(waist) (disc)”, which means “lumbar herniated intervertebral disc”, are considered as a disease.
- Symptoms are usually nouns but can be the combination of adjective and symptom when the expression is very common. For example, “(benumbed) (symptom)”.
- Body tissues such as muscle, ligament or bone are not the target but organs such as stomach, liver, or brain are because they can be the location of a symptom.

Table 3 shows the characteristics of the annotated data. The number of annotated unique terms and that of total annotated terms are given. The annotated dataset will be available on request.^2^

**Table 3:** Characteristics of the annotated diagnosis data

	**DZ**	**SX**	**BP**
# annotated unique entities	297	228	199
# annotated entities	915	1,267	1,010

### Tokenization and labeling for BERT model

**Fig. 4:**
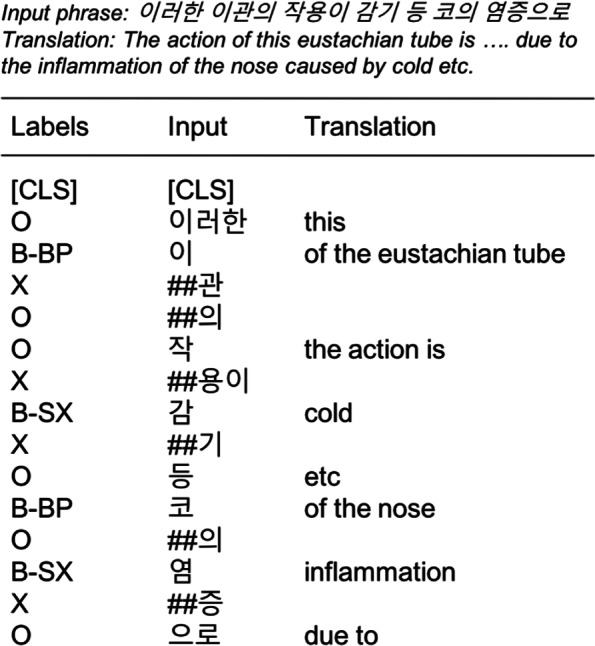
An example of tokenized sentence in diagnosis dataset. Each column corresponds to the output labels, input tokens, and the translation of input in English

In BERT, WordPiece tokenization and three different embeddings are used to represent input tokens. After a punctuation splitting and whitespace tokenization, WordPiece tokenization separates words into different sub-words as explained in the previous section. Figure 4 shows an example of a tokenized sentence in our dataset. The translation in the table is done in word-level separated by whitespaces regardless of the grammatical order. It is because the one-to-one translation from Korean token to English is not possible.

There are two symptoms and two body parts to be detected in the example. The first symptom is ‘(cold)’, which is tokenized as ‘’ and ‘##’. Its first token is annotated as ‘B-SX’ according to the IOB (Inside-Outside-Beginning) tagging and the remainder is annotated as ‘X’. The second symptom, ‘(inflammation)’ is also annotated in the same way.

In the case of the body parts, the annotation is a little different. The first body part, ‘(eustachian tube)’ is originally attached with a genitive postposition, ‘(of)’. After WordPiece tokenization, each syllable(character) is separated, but the third token becomes ‘##’ because it was a part of the word ‘(of the eustachian tube)’. However, in the annotation, only the tokens ‘’ and ‘’ and ‘’. Therefore, the tag of ‘##’ should be ‘O’ (outside), instead of ‘X’. In the original version of BERT does not consider this kind of postposition processing for Korean. It is the main difference when we label the training set compared to the original BERT.

**Fig. 5:**
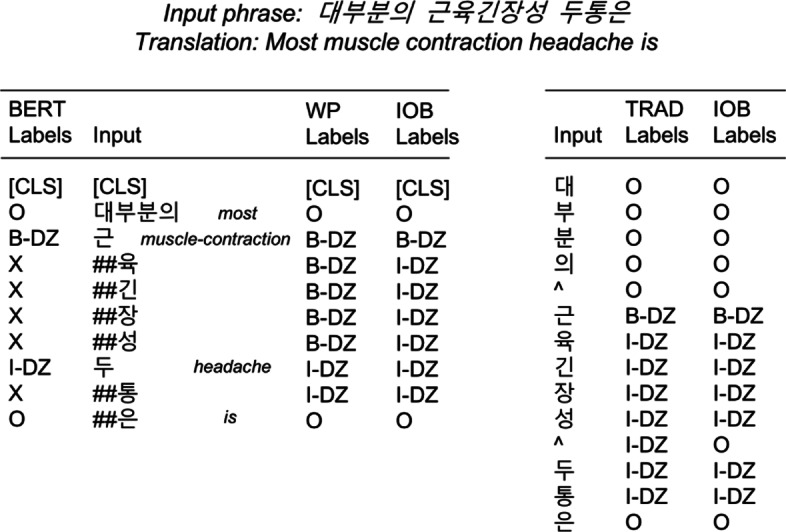
An example of WordPiece input representation with three different labeling strategies (WP, IOB, TRAD) for the evaluation. BERT labels are used for the training, whereas WP and IOB labels are used only for the evaluation

During the evaluation, the tokens attached with ‘X’ tags are ignored to compute word-level frequency in the case of general English NER. However, in Korean, this is not a common approach, especially when using character-level embeddings. Because of the postpositions, more than one label can be attached to a word. Therefore, character-level evaluation can be more exact to verify NER performance for Korean. For the comparison with the existing approaches introducing character embeddings, we propose to additionally use two modified versions of tag representations as shown in Fig. 5 when evaluating the results.

Figure 5 contains two tables representing different labeling strategies for WordPiece embeddings (left) and character embeddings (right) respectively. An example of the input phrase is given on top of the tables. The right side table shows the tokenized input features in character-level and their labels in two different formats. The first and second columns correspond to the typical input tokenization and labeling. Whitespace in the input is replaced by a special character, ‘ ∧’. The foremost token of an entity is annotated as a beginning tag, starting with ‘B’, whereas the others are annotated as ‘I’. When an entity consists of more than a word, the whitespace between words is also annotated as a part of the entity. Many LSTM-based models use this representation as a benchmark.

There is a problem when we use the TRAD labels for the comparison. As BERT does not need to consider whitespaces, there is no label between two separated words in an entity for WordPiece representation. For an effective comparison when evaluating, we propose to use the IOB labels (third column), where the only difference with the TRAD labels is to ignore whitespace’s labels by marking them as ‘O’.

The left table in Fig. 5 shows the WordPiece input representation with three different labeling strategies. The first column corresponds to the default BERT labels that we use for the training. For the token-level evaluation, we define a new labeling strategy, called WP(WordPiece) labeling. The WP labeling strategy simply replaces ‘X’ labels by the precedent token’s label when the token is a part of an entity. In this way, the predicted word boundaries can be correctly evaluated. The IOB labeling strategy is the same as that of the right table. In summary, the default BERT labels are used for the training, whereas the other strategies are used only for the evaluation. The IOB labels are used especially for the comparison of BERT and bi-LSTM-CRF, a benchmark sequence labeling technique.

## Results and discussion

**Fig. 6:**
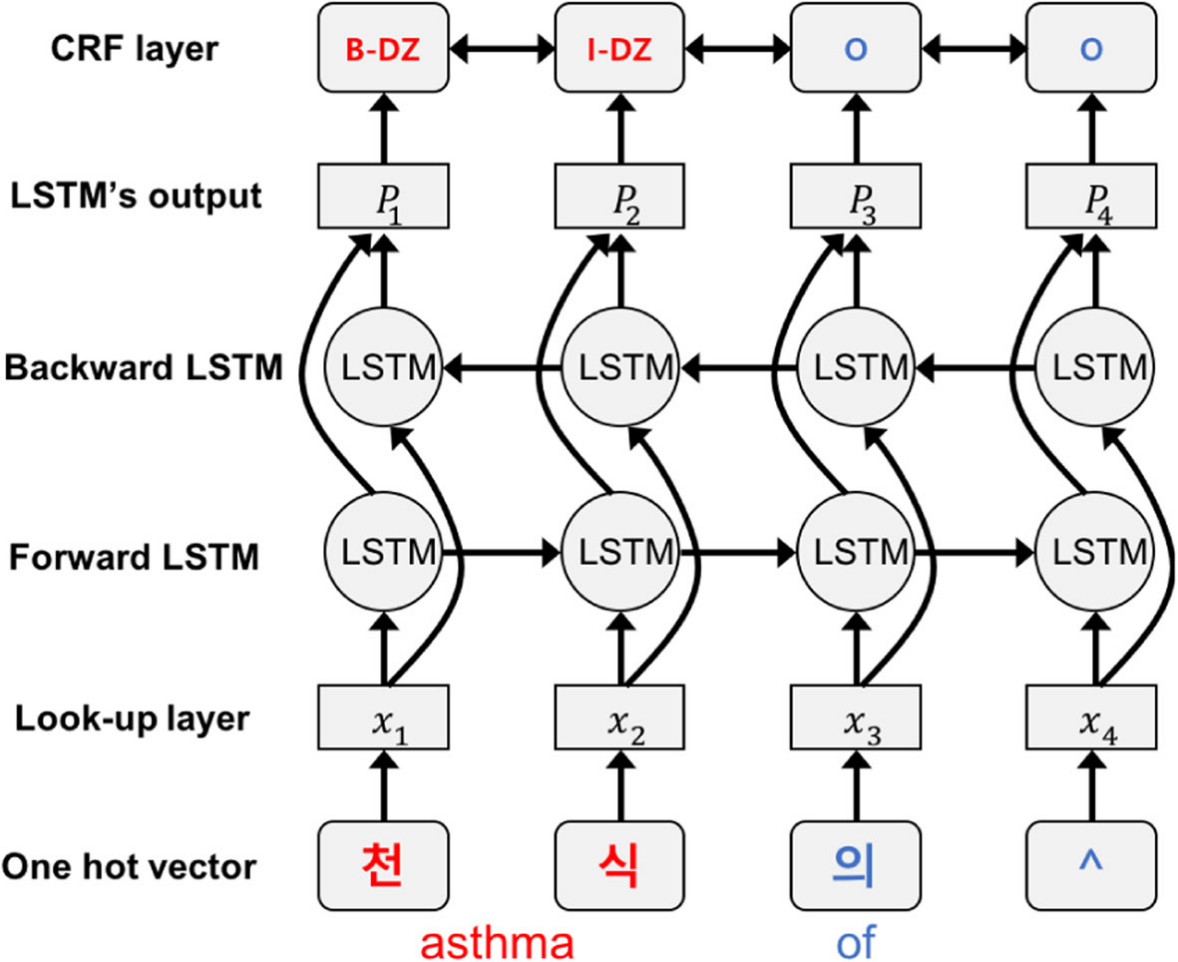
Bi-LSTM-CRF architecture

The goal of the experiments is to verify the effectiveness of BERT for the clinical entity recognition with our novel dataset. However, for the more reliable experiments, we also conducted the experiments on another Korean NER dataset, Exobrain Korean named entity dataset.^3^ It consists of 10,000 sentences with five different entity types: person, location, organization, date, and time. Although the corpus does not aim at medical NER, we expect that this extra experiment would help to justify the effectiveness of BERT for Korean NER. We choose bi-LSTM-CRF as a benchmark model because the model has achieved state-of-the-art performance in Korean NER [20, 22]. The architecture of bi-LSTM-CRF is shown in Fig. 6. It is a bi-directionally connected LSTM variant, which has a CRF layer at the end of the network for the entity recognition.

### Experimental setting

The publicly available TensorFlow version of the official BERT implementation has been used for the experiments. A pre-trained multilingual cased model that is applicable for 104 languages, is selected to deal with Korean. It consists of 12-layer, 768-hidden, 12-heads, and 110M parameters. For the NER task, we need to modify the code to prepare the proper input and output. We modified a publicly available code^4^, which is also based on the original BERT implementation. The experiments are carried out on an NVIDIA P100 16GB GPU.

Maximum sequence length, train batch size, and learning rate are set to default values, 128, 32, and 2e-5 respectively. The number of train epochs is set to 16. We used 5-fold cross validation for the experiments. Therefore, the obtained results are the averaged values on 5 experiments with different train-test splits.

### Experimental results

We first show the NER performance comparison results of BERT and bi-LSTM-CRF on the Exobrain dataset in Table 4. We used the character-level embeddings for the bi-LSTM-CRF model as the example in Fig. 6. The IOB labels introduced in Results and discussion section have been used for the evaluation.

**Table 4:** NER performance comparison results of BERT and bi-LSTM-CRF on the Exobrain dataset

	**BERT (IOB)**		**bi-LSTM-CRF (IOB)**	
	**macro-avg**	**micro-avg**	**macro-avg**	**micro-avg**
Precision	0.93	0.93	0.90	0.89
Recall	0.94	0.94	0.87	0.86
F1	0.94	0.93	0.89	0.88

BERT much outperforms bi-LSTM-CRF in terms of all metrics. The micro-averaged precision, recall, and f1 of BERT are 0.93, 0.94 and 0.93, whereas those of bi-LSTM-CRF are 0.89, 0.86, and 0.88. BERT achieved similar results in terms of macro-averaged metrics also. Considering that BERT does not use the CRF layer in the end, the superiority is impressive.

Table 5 represents the NER performance comparison results of BERT and bi-LSTM-CRF on the diagnosis dataset. The IOB labels are also used for the evaluation. BERT again outperforms bi-LSTM-CRF for all metrics. The micro-averaged precision, recall, and f1 of BERT are 0.83, 0.85 and 0.84, whereas those of bi-LSTM-CRF are 0.82, 0.79, and 0.81 respectively. The recall values of BERT are especially better than that of the other model. It can be interpreted that the trained BERT model could detect out of vocabulary(OOV) words, which have not been observed in the training set, better than bi-LSTM-CRF.

**Table 5:** NER performance comparison results of BERT and bi-LSTM-CRF on the diagnosis dataset

	**BERT (IOB)**		**bi-LSTM-CRF (IOB)**	
	**macro-avg**	**micro-avg**	**macro-avg**	**micro-avg**
Precision	0.82	0.83	0.81	0.82
Recall	0.85	0.85	0.78	0.79
F1	0.83	0.84	0.79	0.81

Now, we show the results of the BERT model evaluated with the labels more suitable for WordPiece embeddings in Table 6. Two previously introduced formats, the original BERT labels, and WP labels are tested. With the BERT labels, we obtain 0.83, 0.84, and 0.84 in micro-averaged precision, recall, and f1. These results are similar to that of BERT evaluated with the IOB labels. However, the macro-averaged metrics produce much worse results than the micro versions at this time. It is because of the imbalance between classes such that few inside tags are found in the training set. Macro average simply takes the mean value of the different classes, whereas the micro version considers the class proportion. Therefore, the worse result of a small class affects a lot the final performance when using the macro versions.

**Table 6:** NER results of BERT on the diagnosis dataset evaluated with BERT labels and WP label

	**BERT labels**		**WP labels**	
	**macro-avg**	**micro-avg**	**macro-avg**	**micro-avg**
Precision	0.72	0.83	0.70	0.82
Recall	0.72	0.84	0.73	0.84
F1	0.72	0.84	0.72	0.83

We obtain similar results using WP labels. The evaluation results with BERT labels are slightly better but statistically insignificant. It means that the trained BERT model successfully split a word at the exact word boundary when inferring.

To verify more in detail the result, we show the detailed performance for each entity-tag in Table 7. In the cases of the BERT labels and WP labels, while the disease tags show good results, the inside tags of the symptom and body part show much worse results than the others. The f1 score for I-DZ is 0.83 whereas that of I-SX and I-BP are 0.55 and 0.39 respectively. The main reason is the imbalance among tags. Many disease names consist of more than a word whereas symptoms and body parts usually consist of a word. This phenomenon cannot be captured using IOB labels because the inside tags are attached to from the second character (syllable) of an entity. Therefore, the IOB labeling strategy is not appropriate for an exact evaluation.

**Table 7:** Detailed evaluation result with BERT for the diagnosis dataset

	**BERT labels**			**WP labels**			**IOB labels**		
	**precision**	**recall**	**f1 score**	**precision**	**recall**	**f1 score**	**precision**	**recall**	**f1 score**
B-DZ	0.85	0.88	0.87	0.85	0.89	0.87	0.85	0.88	0.87
I-DZ	0.83	0.82	0.82	0.83	0.84	0.83	0.85	0.88	0.87
B-SX	0.86	0.85	0.86	0.86	0.84	0.84	0.86	0.85	0.86
I-SX	0.53	0.54	0.54	0.54	0.55	0.55	0.83	0.81	0.82
B-BP	0.83	0.86	0.84	0.78	0.83	0.81	0.83	0.86	0.84
I-BP	0.38	0.39	0.39	0.36	0.42	0.39	0.72	0.79	0.75

### Transfer learning

In addition to the previous experiments, we also try applying the trained model to the question data. As the writing style of question set is significantly different from that of answer set, we suppose that two sets are from different domains. Therefore, this additional experiment is a kind of transfer learning, more specifically domain adaptation. The source domain is the answer set and the target domain is question set. For the evaluation of this experiment, we also annotated the question set.

Table 8 represents the detailed result of transfer learning. For the entity types disease and body part, we obtain a slightly worse performance than the standard learning. F1 scores of B-DZ, I-DZ, B-BP, and I-BP are 0.84, 0.85, 0.81 and 0.39 respectively. It means that disease and body part of user questions are automatically extractable using the BERT model trained with the diagnosis (answer) dataset.

**Table 8:** Transfer learning result tested with the question data using BERT model trained with the diagnosis (answer) dataset

	**WP labels**		
	**precision**	**recall**	**f1 score**
B-DZ	0.87	0.81	0.84
I-DZ	0.84	0.85	0.85
B-SX	0.78	0.52	0.63
I-SX	0.19	0.07	0.10
B-BP	0.81	0.81	0.81
I-BP	0.69	0.26	0.37
macro-avg	0.70	0.55	0.62
micro-avg	0.81	0.67	0.73

On the other hand, the performance in terms of symptom is considerably worse than the others. The recall values are particularly bad such as 0.52 for B-SX and 0.07 for B-BP. We assume that this result comes from the difference between the two sets when representing symptoms. The question set has a lot of mimetic words such as ‘pitapat’ and ‘pricking’. It has also many adjectives symptoms such as ‘dizzy’ and ‘stabbing’ whereas the answer set’s symptoms are mainly nouns. This difference was likely to influence performance. Therefore we cannot apply this simple transfer learning when detecting symptoms from user questions.

## Conclusions

In this paper, we show that the recently developed Bidirectional Encoder Representations from Transformers (BERT) model is effective for the Korean clinical entity recognition. A new dataset for the clinical entity recognition and a standard NER dataset have been tested to verify the effectiveness. The result suggests that the WordPiece tokenization is sufficient in the BERT framework to obtain a state-of-the-art result for the entity recognition in the Korean language. For future work, we are interested in enhancing the prediction performance by combing the core BERT framework with a modified end layer of the network. Then we will move into the next step of the diagnosis system, the extraction of question intent and the construction of the dialogue manager.

## Data Availability

The datasets generated and analysed during the current study are available on request in the GitHub repository, https://github.com/labihem/BERT-NER-KO.

## Abbreviations

BERT: Bidirectional encoder representations from transformers
EHRs: Electronic health records
LSTM: Long-short term memory
CRF: Conditional random fields
bi-LSTM-CRF: Bidirectional LSTM-CRF
IOB: Inside-outside-beginning
OOV: Out of vocabulary
CNN: Convolutional neural network
NER: Named entity recognition
ELMo: Embeddings from language models
GPT: Generative pre-training transformer
NLP: Natural language processing
LM: Language model
DZ: Disease
SX: Symptom
BP: Body part

## Footnotes

1 https://kin.naver.com

2 https://github.com/labihem/BERT-NER-KO

3 http://aiopen.etri.re.kr/

4 https://github.com/kyzhouhzau/BERT-NER
